# Walking away from type 2 diabetes: trial protocol of a cluster randomised controlled trial evaluating a structured education programme in those at high risk of developing type 2 diabetes

**DOI:** 10.1186/1471-2296-13-46

**Published:** 2012-05-29

**Authors:** Thomas Yates, Melanie J Davies, Joe Henson, Jacqui Troughton, Charlotte Edwardson, Laura J Gray, Kamlesh Khunti

**Affiliations:** 1 Department of Cardiovascular Sciences, University of Leicester, Leicester, UK; 2 Diabetes Research, University Hospitals of Leicester NHS Trust, Leicester, UK; 3 Department of Health Sciences, University of Leicester, Leicester, UK; 4 Leicester Diabetes Center, Leicester General Hospital, LE5 4PW, Leicester, UK; 5 NIHR Leicester-Loughborough Diet, Lifestyle and Physical Activity Biomedical Research Unit, Leicester, UK

**Keywords:** Pedometer, Physical activity, Primary care, Prevention, Type 2 diabetes, Walking

## Abstract

**Background:**

The prevention of type 2 diabetes is a recognised health care priority globally. Within the United Kingdom, there is a lack of research investigating optimal methods of translating diabetes prevention programmes, based on the promotion of a healthy lifestyle, into routine primary care. This study aims to establish the behavioural and clinical effectiveness of a structured educational programme designed to target perceptions and knowledge of diabetes risk and promote a healthily lifestyle, particularly increased walking activity, in a multi-ethnic population at a high risk of developing type 2 diabetes.

**Design:**

Cluster randomised controlled trial undertaken at the level of primary care practices. Follow-up will be conducted at 12, 24 and 36 months. The primary outcome is change in objectively measured ambulatory activity. Secondary outcomes include progression to type 2 diabetes, biochemical variables (including fasting glucose, 2-h glucose, HbA1c and lipids), anthropometric variables, quality of life and depression.

**Methods:**

10 primary care practices will be recruited to the study (5 intervention, 5 control). Within each practice, individuals at high risk of impaired glucose regulation will be identified using an automated version of the Leicester Risk Assessment tool. Individuals scoring within the 90^th^ percentile in each practice will be invited to take part in the study. Practices will be assigned to either the control group (advice leaflet) or the intervention group, in which participants will be invited to attend a 3 hour structured educational programme designed to promote physical activity and a healthy lifestyle. Participants in the intervention practices will also be invited to attend annual group-based maintenance workshops and will receive telephone contact halfway between annual sessions. The study will run from 2010–2014.

**Discussion:**

This study will provide new evidence surrounding the long-term effectiveness of a diabetes prevention programme run within routine primary care in the United Kingdom.

**Trial Registration:**

ClinicalTrials.Gov identifier: *NCT00941954*

## Background

### Overview

The high prevalence of type 2 diabetes mellitus witnessed in the United Kingdom and globally represents one of the greatest public health challenges in the 21^st^ century [1]. Currently, the treatment of diabetes accounts for 7–14% of total health care spending across low to high income regions of the globe and this is projected to increase in the future [2]. This has prompted international and national health care organisations to focus on prevention through targeted recommendations and policy. In the United Kingdom, the NHS health checks programme is aimed at screening all individuals between 40 to 74 years of age for vascular and metabolic disease risk and then treating high risk individuals accordingly [3]. Preventing type 2 diabetes is one of the central aims of this programme. However in the UK, translational research has lagged behind policy change and there has been a lack of diabetes prevention programmes specifically developed for, and evaluated in, routine health care settings. Therefore research programmes are urgently needed to address this need.

### Physical activity and lifestyle in the prevention of type 2 diabetes

Type 2 diabetes is widely considered a lifestyle disease because of its strong links to deleterious lifestyle practices associated with industrialisation. For example, it has been shown that 80–90% of all cases of type 2 diabetes could be prevented through a healthy lifestyle [4,5]. Furthermore, randomised controlled trials across diverse countries and populations have shown that the risk of progressing to type 2 diabetes in high risk populations can be reduced by up to 60% in those receiving lifestyle interventions aimed at promoting moderate- to vigorous-intensity physical activity along with a healthy diet, and weight loss/maintenance [6]. The evidence underpinning the link between physical activity and glucose regulation is particularly compelling because it is supported by the full spectrum of evidence needed to infer causality, from observational research [7], to experimental mechanistic investigation [8], through to randomised controlled trials [6,9]. The general importance of physical activity to health was recently highlighted by the World Health Organization who now rank physical inactivity as the fourth leading cause of premature mortality globally ahead of both obesity and dietary factors [1].

### Translation of research into practice

Lifestyle diabetes programmes with proven effectiveness have used resource-intensive behaviour change strategies involving multiple one-to-one patient contacts which are incompatible with routine healthcare settings given competing health care needs and resource and infrastructure limitations [10]. Therefore, diabetes prevention pathways that are tailored to national and regional health care systems need to be developed and evaluated. Over the last decade, several countries including the United States, Finland, Germany and Australia have met this challenge by developing and evaluating diabetes prevention pathways designed for routine clinical care [11]. Although undertaken in diverse settings, these translational programmes have tended to centre on group-based health promotion programmes aimed at replicating the behavioural goals of gold standard research programmes [11]. However, despite these international advances, there has been limited translational research in the United Kingdom. This has consequently limited the ability of primary care providers to commission robust evidence-based diabetes prevention programmes. In response to this need, we developed and evaluated a brief structured education programme designed to promote increased walking activity and improved glucose regulation in those at a high risk of type 2 diabetes as identified through impaired glucose tolerance. This programmes was found to be highly effective at promoting long-term changes to health behaviour and improvements in glucose regulation [9,12]. The programme was specially tailored to the needs and infrastructure available within primary care; this was achieved through modelling the structure and educational philosophy on an established and nationally available self-management programme for those with diagnosed type 2 diabetes [13]. Therefore the effectiveness of this approach at promoting health behaviour and self-management skills in the prevention of diabetes needs to be established within a routine primary care setting.

### Risk identification

In order to identify those who should be referred into a prevention programme, it is also important to design and evaluate pragmatic methods of identifying those with a high risk of developing type 2 diabetes which can be integrated into routine health care systems. Evaluated diabetes prevention programmes have recruited those with impaired glucose tolerance, commonly referred to as prediabetes, identified through an oral glucose tolerance test (OGTT) [6,11]. This test incurs a substantial burden to both health care professionals and patients and is not routinely conducted or recommended as a method of assessing diabetes risk. Risk scores, either in conjunction with, or instead of, biochemical testing are widely acknowledged as a pragmatic and effective way of identifying risk status and their use is promoted by European level guidance on the prevention of type 2 diabetes [14]. However, there is limited evidence from randomised controlled trials to establish the clinical effectiveness of intervening in those identified by a risk score who are likely to have a lower absolute risk of developing diabetes and represent different characteristics compared to those identified with impaired glucose tolerance.

### Study aim

The aim of the study is to establish whether a pragmatic structured education programme with proven efficacy can promote health behaviour and improve metabolic health in individuals at high risk of type 2 diabetes identified through a validated risk score in primary care.

### Primary objective

· To investigate whether a lifestyle intervention programme, based on a brief pragmatic education programme with minimal ongoing support, can promote sustained long-term increases in physical activity in those identified with a high risk of type 2 diabetes in a primary health care setting.

### Secondary objectives

· To investigate the impact of a lifestyle intervention programme on glucose regulation, progression to type 2 diabetes and conventional cardiovascular risk markers in those identified with a high risk of type 2 diabetes through a validated risk score

· To investigate the impact of a lifestyle intervention programme on measures of quality of life and depression in those with a high risk of type 2 diabetes

· To investigate the natural history of progression to type 2 diabetes over a three year period in high risk individuals identified through risk score technology.

## Methods

### Study design

**Figure 1:**
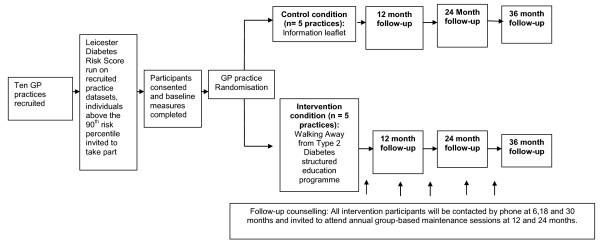
Design and flowchart for the walking away from type 2 diabetes study.

This study is a clustered randomised controlled trial. Randomization will be conducted at the level of the GP practice by a trained individual who is independent of the study team. Practices were randomised (1:1) to receive control conditions or a structured education programme. A blocked design stratified for practice size was used. The study is designed to adhere to internationally recognized criteria for developing complex interventions [15] and for undertaking and reporting cluster randomised controlled trials [16]. Follow-up will be conducted at 12, 24 and 36 months in order to establish the longer-term effectiveness of the programme and determine the natural history of progression to type 2 diabetes over three years in a high risk UK cohort identified through a risk score in primary care. See Figure 1 for a flow-diagram of the study design. 

### Sample size

This study was powered to detect a difference in change in ambulatory activity of 2000 steps per day between groups; a difference of this magnitude reflects the primary behaviour change aim of the intervention programme and is consistent with the national physical activity recommendations and the intervention effect observed in the proof of concept study that preceded this trial [9,17]. Previous studies with a follow-up of between 3 to 12 months have reported a standard deviation of change in ambulatory activity of 2500–4000 steps per day in relevant populations [9,18-20]. Therefore, in order to detect a difference of 2000 steps per day between groups, assuming a standard deviation of 4000 steps per day, a power of 90%, a significance of 0.05, a cluster size of 90 (see section titled Participants below) and an intracluster correlation coefficient of 0.02 [21], we require a minimum of 8 clusters. In order to account for potential dropout at the practice level and comply with guidance from the Medical Research Council for minimum cluster numbers [22], 10 GP practice clusters will be recruited to this trial.

This sample size will also have sufficient power to enable clinically significant differences to be detected between groups for change in important secondary outcomes. For example, based on a power of 80%, a significance of 0.05 and a intracluster correlation coefficient of 0.02 [23], the above sample size will allow for a reduction in 2-h glucose of 0.8 mmol/l (based on a standard deviation of 2 mmol/l standard deviation [24]) and a reduction in HbA1c of 0.2% (based on a standard deviation a standard deviation of 0.5% [24,25]) to be detected.

### Participants

Ten GP practices will be recruited from the Leicestershire region (city and county primary care trusts). Initial contact with each GP practice will be through a letter of invitation to take part. All recruited practices will have an induction visit from the study co-ordinator who will provided training and support for the administrative staff in each of the study sites. Eligible participants will be identified using an automated version of Leicester Risk Assessment tool developed and validated by our group [26]. The automated risk score uses the Morbidity, Information Query and Export Syntax (MIQUEST) programme, designed to extract data from computerized medical records in primary care, to analyse data from six variables (age, sex, ethnicity, BMI, family history of type 2 diabetes, antihypertensive medication status) that are commonly held on local PCT databases. By assigning pre-validated weighting to each variable, individuals are ranked by their risk of undiagnosed impaired glucose regulation or type 2 diabetes with a higher score indicating higher risk. The risk score is calculated as follows:

Risk factor Score = 0.0407 x age

+  0.296 (if male, no change in female)

+ 0.934 (ethnicity, as a practice proportion of south Asians)

+ 0.0859 x BMI

+ 0.440 (if family history of type 2 diabetes, no change otherwise)

+ 0.374 (if on antihypertensive medication, no change otherwise)

In this study, high risk individuals will be classified as those above the 90^th^ percentile of the calculated risk score. Eligible individuals will be sent a letter of invitation, a patient information sheet and stamped addressed envelope by a member of their GP practice. Contacted individuals will be requested to return a reply slip confirming or declining their interest in taking part; those who wish to take part will be contacted by the study team where a baseline consent appointment is arranged. 

Individuals will be excluded from the study if they have an existing diagnosis of type 2 diabetes or are diagnosed with type 2 diabetes at baseline, are taking steroids or are unable to speak English; our experience suggests this approach does not limit recruitment and is able to produce a study population that fully reflects the ethnic makeup of the local population.

Based on a typical GP practice of 4500 adults without known type 2 diabetes, 450 will be eligible for inclusion into the study based on the risk score. Of these we anticipated that 330 (75%) will meet the initial inclusion criteria and be invited to take part in the study. Around 100 (30%) are then expected to consent to take part, of which 10 (10%) will have screen detected type 2 diabetes and therefore excluded at baseline; therefore we anticipate the average cluster size to be 90 individuals (see sample size calculation).

Those diagnosed with diabetes over the course of the study will be informed of their diagnosis and referred back to their health care team for standard care. Progression to type 2 diabetes will be included as a secondary outcome.

Figure 1 shows the flow of the participants through the study.

### Setting

The study is co-ordinated from the University Hospitals of Leicester. However, clinical measurement sessions and the educational programmes will be run in a variety of different locations within the Leicestershire area, including: hospital, primary care and community settings. Locations that are as near as possible to the recruited GP practices will be identified, including within the practice itself where possible.

### Treatment regimens

#### Control

Control subjects will receive a booklet detailing information on risk factors for type 2 diabetes and how physical activity and lifestyle change can be used to prevent or delay the disease. The leaflet addresses factors around type 2 diabetes risk using the five domains (causes, consequences, identity, control/treatment, and timeline) highlighted by Leventhal’s common sense model [27].

#### Intervention

The intervention group will be offered the Walking Away from Type 2 Diabetes group-based structured educational programme. This programme is based on the content and behaviour change techniques of the successful Prediabetes Risk Education and Physical Activity Recommendation and Encouragement (PREPARE) programme; a full description of the rationale, development and efficacy of which can be found elsewhere [9,28]. Walking Away will be delivered to 6–10 individuals by two trained educators over 3 hours and is primarily designed to promote walking activity by targeting perceptions and knowledge of impaired glucose tolerance and physical activity self-efficacy as well as promoting self-regulatory skills such as goal-setting strategies, self-monitoring, and relapse prevention (identifying and addressing barriers to change). Self-regulation is based around pedometer use, which has been demonstrated to be crucial to the success of the programme [9]. Specifically, those taking less than 6000 steps at baseline are encouraged to increase their activity levels by at least 3000 steps per day, equivalent to around 30 min of walking [29]. Those achieving more than 6000 steps per day are encouraged to try to reach at least 9000 steps per day, an amount that is likely to include 30 min of walking activity in addition to usual daily activity [29]. Those achieving more than 9000 steps per day are encouraged to at least maintain their current activity levels and informed that health benefits could be achieved by increasing their activity levels further. Goal attainment is encouraged through the use of proximal objectives. Participants are enabled to set an action plan detailing where, when and how their first proximal goal will be reached and encouraged to repeat this process for each new proximal goal. Participants are encouraged to wear their pedometer on a daily basis and to self-monitor their ambulatory activity using a steps-per-day log. Along with physical activity, the programme has a specific section dedicated to healthy dietary practices, particularly around the identification and substitution of food items high in saturated fat.

The programme content is underpinned by an integrated theoretical framework, focused on linking motivational and volitional determinants of health behaviour. The framework is based on mutually complementary health behaviour theories, including Bandura’s social cognitive theory [30], Gollwitzer’s implementation intentions [31], Leventhal’s common sense model [27], and Chaiken’s dual process theory [32] and was informed by, and modelled on, the person-centred philosophy and learning techniques developed for the DESMOND programme [13]; a nationally available self-management programme for those with type 2 diabetes run within half of all PCTs nationally. The programme was developed inline with the Medical Research Council’s Framework for Complex Interventions to Improve Health, which are internationally recognised criteria for guiding the development and evaluation of health behaviour change programmes [15,33].

The walking Away programme was adapted for a broader range of high risk individuals, such as those identified through a risk score, in order to make it suitable for translation into usual healthcare practice. This necessitated a shift in focus from the PREPARE programme, which targeted illness perceptions around prediabetes, to those associated with a more generic high risk label. In order to meet this need, an additional section was added to the programme that was aimed at communicating the meaning of risk and risk status. Modifications to the PREPARE programme were undertaken following an established cyclic process that involved training a pool of educators and then piloting the programme to a reprehensive patient group whereupon patient and educator level feedback were collected and the programme revised accordingly. This process was repeated twice.

Table 1 provides an overview of the programme content and structure.

**Table 1:** Outline of the Walking Away programme

**Module**	**Main aims**	**Example activity**	**Theoretical underpinning**	**Time weighting**
Introduction	Welcome/housekeeping			5 minutes
Patient Story	·Give participants a chance to share their knowledge and perceptions of being identified as ‘at risk’ of type 2 diabetes and highlight any concerns they may want the programme to address.	·Participants are asked to share their story, how they were diagnosed as being ‘at risk’ of developing type 2 diabetes and their current knowledge of being ‘at risk’	·Common Sense Model [27]	25minutes
Professional story	Use simple non-technical language, analogies, visual aids and open questions to provide participants with:	·Individuals are helped to plot their individual risk (fasting and 2 hour blood glucose levels, cholesterol and blood pressure levels - assessed at baseline)	·Common Sense Model [27]	35 minutes
			·Dual Process Theory [32]	
	·An overview of healthy glucose metabolism			
			·Social Cognitive Theory [30]	
	·The aetiology of diabetes			
	·An overview of the macrovascular complications associated with being ‘at risk’ of type 2 diabetes.			
Risk story	·The meaning and assessment of risk in the context of developing type 2 diabetes	·Participants are supported to plot their own risk factors onto a risk chart to work out their individual risk areas.	·Dual Process Theory [32]	25 minutes
			·Social Cognitive Theory [30]	
	·Explore personal risk of developing type 2 diabetes			
Break	Refreshments and informal discussion			10 minutes
Physical activity	Use simple non-technical language, analogies, visual aids and open questions to help participants:	·Individuals are helped to plot their individual steps per day scores (assessed at baseline)	·Social Cognitive Theory [30]	55 minutes
			·Implementation Intentions [31]	
	·Identify how physical activity improves glucose control;			
	·Understand the current physical activity recommendations	·Participants are provided with a physical activity diary and encouraged to set their first action plan.		
	·Explore options for incorporating physical activity (primarily walking) into everyday life			
	·Identify barriers to exercise			
	·Form action plans			
	·Use their provided physical activity diaries			
	·Set personal goals (based on baseline pedometer counts)			
			·Dual Process Theory [32]	
Diet	·Give participants an accurate understanding of the link between dietary macro-nutrients and metabolic dysfunction	·Participants are asked to group models of fats and oils into saturated, polyunsaturated and monounsaturated categories.	·Social Cognitive Theory [30]	20 minutes
			·Dual Process Theory [32]	
Conclusion	Questions and future care	Sign-post to locally available groups/programmes		5 minutes

#### Educator recruitment, training and quality assurance

A pool of around 10 educators will be recruited from primary and secondary care and the community. Those recruited from a health care setting will be registered health care professionals and those from the community will be required to process a degree in a health science or be employed within a relevant vocation (i.e. gym instructor). Educator training will consist of two full core days followed by regular ongoing support. Training will be provided through fully accredited national trainers for structured education programmes hosted by the DESMOND collaborative. In order to ensure that the programme philosophy and content are adhered to, a quality assurance procedure was also developed based on established criteria used within the DESMOND programme [34]. This involves an assessor sitting quietly and unobtrusively at the back of the room, with a CD playing into a headphone whilst observing the programme. The CD is silent, except for a beep sounding every 10 s. When the beep sounds, the assessor indicates on a response sheet who is talking at that point (educator or participant), with other activity classed as ‘miscellaneous’ (silence, laughter or multiple conversations during learning activities) [34]. At the same time the assessor fills in a prompt sheet indicating whether or not key learning points within each module were covered. All quality-assured educators receive feedback from their assessor and key goals and action plans are developed in order to help the educator improve their performance.

#### Follow-up support

After the participant’s annual clinical measurement session, all individuals in the intervention group will be invited to attend annual group-based sessions at 12 and 24 months in order to establish the impact of minimal maintenance support on long-term outcomes. These follow-up programmes will be designed to help participants interpret and analyse their annual biochemical and anthropometric follow-up data, review progress and goals and respond to issues, queries and barriers; the main objectives of the programme will also be reinforced. Each follow-up programme will last 2–3 h and will be conducted by a trained educator. All participants will also receive telephone contact between annual sessions in order help participants maintain their motivation.

### Primary outcome

#### Physical activity

The primary end point will be ambulatory activity assessed by tri-axial accelerometers (GT3X, Actigraph, FL, USA). This outcome reflects the overall aim of the intervention and is consistent with the fact that walking activity is the preferred choice of activity within the population. Participants are asked to wear the accelerometer, fitted on their trunks (placed on right anterior axillary line) with a waistband, for seven consecutive days during waking hours. These accelerometers are one of the most extensively validated and accurate on the market and are the only commercially available accelerometers to correlate with energy expenditure as measured by double-labelled water [35].

Overall physical activity as defined by total body movement (counts per day), and time in sedentary, light-, moderate- and vigorous-intensity physical activity will also be assessed by accelerometer and determined by validated counts-per-minute cut-points [36].

Self-reported physical activity will also be measured using the short last-seven-days self-administered format of the International Physical Activity Questionnaire (IPAQ). This questionnaire provides a comprehensive measure of walking and other moderate- to vigorous-intensity activities carried out for more than 10 continuous minutes at work, in the home, as transport and during leisure time. IPAQ has been shown to have reasonable validity compared to accelerometer data (ρ ~ 0.4) and test-retest reliability (ρ ~ 0.7) in the United Kingdom when used as a measure of total moderate- to vigorous-intensity physical activity [37]. For this study, IPAQ will be used to measure total walking activity as well as an overall measure of moderate- to vigorous-intensity physical activity accumulated over all contexts.

All physical activity outcomes will be measured at baseline and 12, 24 and 36 months.

### Secondary outcomes

#### Biochemical variables

This study will measure relevant markers of metabolic and renal health including fasting and 2-h post challenge glucose, HbA1c, lipid profile, liver function tests (albumin, total bilirubin, alkaline phosphatase [ALP], alanine transaminase [ALT]), urea and electrolytes (sodium, potassium, creatinine). Analysis is conducted in the same laboratory located within Leicester Royal Infirmary, UK, using stable methodology standardized to external quality assurance reference values.

Participants will be invited to attend each clinical measurement session after a 12-h fast and 24 h of avoiding vigorous intensity exercise. In concordance with WHO recommendations, those who have a fasting or 2-h blood glucose level in the diabetes range at any clinical measurement session will be called back for a confirmatory oral glucose tolerance test [38].

All venepuncture and OGTT timings will be undertaken by trained phlebotomists who are not part of the scientific advisory team for this study and who are blinded to treatment allocation. All biochemical analysis will also be conducted blinded to treatment group.

#### Anthropometric and demographic variables

Arterial blood pressure will be measured in the sitting position (Omron, Healthcare, Henfield, UK); three measurements will be obtained and the average of the last two measurements will be used. Body weight and body fat percentage (Tanita TBE 611, Tanita, West Drayton, UK), waist circumference (midpoint between the lower costal margin and iliac crest) and height will also be measured, to the nearest 0.1 kg, 0.5% and 0.5 cm respectively. Information on current smoking status, medication history, and ethnicity is obtained by self-report. Social deprivation will be determined by assigning an Index of Multiple Deprivation (IMD) score to participant postcodes [39]. IMD scores are publicly available continuous measures of compound social and material deprivation which are calculated using a variety of data including current income, employment, health, education, and housing.

#### Diet

Diet will be measured using the Dietary Instrument for Nutrition Education (DINE) food frequency questionnaire, which was designed as a method of measuring fibre, fat and unsaturated fat intake in primary care [40]. The DINE food frequency questionnaire has been shown to have reasonable validity when assessed against food records (0.45 < r < 0.51) [40,41].

#### Health related quality of life

Health related quality of life will be measured by the 15D instrument, which can be used as a profile or a single index score utility measure [42]. It consists of 15 dimensions: mobility, vision, hearing, breathing, sleeping, eating, speech, elimination, usual activities, mental function, discomfort and symptoms, depression, distress, vitality and sexual activity. Each dimension is divided into five ordinal levels, by which more or less of the attribute can be distinguished. The valuation system of the 15D is based on an application of the multi-attribute utility theory. A difference of ≥0.03 in the 15D single index score utility score is considered clinically important [42].

#### Perceptions and perceived knowledge of diabetes risk

Perceptions and perceived knowledge of diabetes risk will be measured with the validated brief illness perceptions questionnaire [43]. This eight item instrument uses an 11 point Likert scale (0 = no effect, 10 = complete effect) to measure five cognitive illness representations (consequences, timeline, personal control, treatment control, and identity), two emotional representations (concern and emotion) and illness comprehensibility (perceived knowledge). The brief illness perception question provides a practical and comprehensive measurement of determinants identified in Leventhal’s common sense model [27], one of the key theoretical models underpinning the content and structure of the education programme.

#### Depression and anxiety

Depression and anxiety will be measured with the Hospital Anxiety and Depression Scale (HADS) [44]. HADS consists of 14 items, with two sub-scales measuring symptoms of depression and symptoms of anxiety. HADS is widely used and has been shown to perform well in primary care [45].

### Data analysis

The study will be reported according to the internationally recognised CONSORT statement for the reporting of cluster randomised control trials [16]. Those withdrawn from the study due to a diagnosis of type 2 diabetes will have their last observation carried forward. Sensitivity analysis, using multiple imputation, will be used to assess the effect of those missing to follow-up. Random effects regression models, controlled for baseline value and taking into account cluster, will be used to look at the difference between groups in change from baseline in continuous outcome measures after checking for normality and applying transformation techniques where necessary. Extreme outliers (more than 4 standard deviations from the mean) will be removed from the analysis. Survival curves will be calculated to estimate the cumulative incidence of diabetes. The difference in incidence of diabetes in the groups will be tested using the two-sided log-rank test. Statistical significance will be assessed at the 5% level and all analysis will be 2-sided.

Interim analysis will be conducted and reported at 12 months.

In concordance with other physical activity randomised controlled trials undertaken by our group and others [46,47], we will undertake pooled analysis of the study cohort to determine, through regression analysis techniques, the extent to which change in physical activity is associated with change in key biochemical and anthropometric variables; this will help provide additional information quantifying the strength of the association between physical activity and metabolic health in a population with a high risk of type 2 diabetes; any such analysis will be reported with the caveats inherent in undertaking additional pooled cohort analysis within the context of a randomised controlled trial.

### Current status

This study gained full ethical and governance approvals from the Nottingham Research Ethics Committee 2 and the Leicestershire, Northamptonshire and Rutland Comprehensive Local Research network in April 2009. Recruitment of patients was initiated in 2010.

## Discussion

To our knowledge this will be the first study in the United Kingdom to establish the long-term effectiveness of an intervention programme designed to promote lifestyle change in those with a high risk of type 2 diabetes identified using a risk score within primary care. Whilst both the effectiveness and cost-effectiveness of lifestyle intervention programmes at preventing type 2 diabetes have been established [6,48], there has been a lack of translational research aimed at developing and evaluating a diabetes prevention programmes specifically designed for a routine health care setting in the UK. This has limited the ability of health care commissioners and policy makers to make informed evidence-based decisions regarding the implementation of regional and national diabetes prevention programmmes. This study will help address this limitation.

## Competing interests

The authors declare that they have no competing interests.

## Authors’ contributions

TY wrote the manuscript and contributed to the conception and design of the study. MJD, JT and KK contributed to the conception and design of the study and revised the manuscript for important intellectual content. JH, CE and LG contributed to the design of the study and revised the manuscript for important intellectual content. All authors read and approved the final manuscript

## Pre-publication history

The pre-publication history for this paper can be accessed here:

http://www.biomedcentral.com/1471-2296/13/46/prepub
